# Nitric oxide mediated the effects of nebivolol in cardiorenal syndrome

**DOI:** 10.22038/ijbms.2019.37400.8927

**Published:** 2019-11

**Authors:** Guldem Mercanoglu, Onder Semen

**Affiliations:** 1 University of Health Sciences, Faculty of Pharmacy, Department of Pharmacology, Istanbul, Turkey; 2 Istanbul University, Istanbul Medical Faculty, Department of Pathology, Istanbul, Turkey

**Keywords:** Cardiorenal syndrome, Myocardial infarction, Nebivolol, Nitric oxide, Nitrosative damage

## Abstract

**Objective(s):**

Despite several proposed mechanisms for the pathophysiology of cardiorenal syndrome (CRS), the exact mechanism remains unclear. Nitrosative stress has been argued as a key mechanism recently. Nebivolol is a beta-blocker with nitric oxide (NO)-releasing effect. In the present study, NO-mediated effects of two different treatment regimes of nebivolol in CRS were studied.

**Materials and Methods:**

Rats were divided into: sham-operated (sham-control), myocardial infarction (MI)-induced, (MI-control) early nebivolol-treated (MI-neb1) and late nebivolol-treated (Mı-neb2) groups. The effects of nebivolol were assessed both in the early and late period of MI by histologic, hemodynamic and biologic studies.

**Results:**

Developed MI model was in line with the heart failure with preserved ejection fraction. Focal and total tubular damage findings were observed in MI-control group both in early and late period of MI. In parallel, subclinical functional damage was transformed into chronic renal dysfunction in this group. Increased inducible nitric oxide synthase (iNOS) and endothelial NOS (eNOS) together with decreased neuronal NOS (nNOS) levels were in parallel with the increased inflammation and nitrosative stress biomarkers. Nebivolol effectively prevented both subclinical and clinical nephropathy. There was no statistical difference between the nebivolol treatment regimes.

**Conclusion:**

The beneficial effects of nebivolol were closely related to the reduction of nitrosative damages as well as hemodynamic alterations. The NO-mediated effects were: prevention of nitrosative damage by decreasing iNOS, preservation of nNOS in order to maintain glomerular filtration rate (GFR), and restoration of eNOS in the late period of MI. On contrary to our previous work, early nebivolol administration had a similar effect with delayed administration of nebivolol on CRS.

## Introduction

It is known that heart and kidney diseases affect each other through different mechanisms. Although complex interplay between the heart and kidneys is known from the beginning of the nineteenth century (1), this was recently redefined as cardiorenal syndrome (CRS). CRS can be classified as Type I-IV according to the time frame and order of occurrence (2). While acute kidney injury (AKI) that develops after myocardial infarction (MI) is known as Type I, renal failure that develops in long-term period is known as Type II. Type I CRS is the most frequent (48.2 %) followed by Type II CRS (21.9 %) (3).

Several mechanisms have been studied to explain the pathophysiology of CRS, such as hemodynamic alterations, endothelial dysfunction, oxidative stress, immune activation/inflammation, renin-angiotensin-aldosterone system (RAAS) and sympathetic system activation (4). Although the exact mechanism remains unclear and more than one pathophysiological process might be operative, oxidative stress induced by inflammation has been argued to be an important trigger in this cascade. It is known that nitric oxide (NO) has a key role both in renal and cardiac physiology and pathophysiology. Although the hemodynamic actions of NO have received much attention, a variety of non-hemodynamic actions have been studied recently (5). NO is a weak reactive nitrogen spices (RNS); however, it may induce the production of peroxynitrite (ONO_2_) and other highly oxidizing reactive oxygen species (ROS) (5). The role of NO-mediated oxidative stress has been shown both in the heart and renal failure. Recent findings also suggest that oxidative/nitrosative stress play a role in the pathogenesis of CRS. 

Nebivolol is a beta-blocker agent. Although NO-mediated effects of nebivolol have been demonstrated in different cardiovascular pathologies, these effects on renal dysfunction are unknown. In this study, we aimed to evaluate the NO-mediated effects of two different treatment strategies of nebivolol in the development and progression of CRS. 

## Materials and Methods

***Animals and dose selection***

Sprague-Dawley rats (250-300 gram; 12 weeks old) were divided into 4 groups of 18 each: Sham operated control (sham-control); MI induced control (MI-control); MI induced and immediate intravenous load followed by oral nebivolol-treated group (MI-neb1) and MI induced and oral nebivolol-treated group (MI-neb2)

Nebivolol dose was selected as the minimum beta-blocker dose (characterized by the absence of significant effect on blood pressure and heart rate) in a preliminary dose-response study (using 0.1, 0.5 and 1 milligram/kilogram of nebivolol intravenously (IV)) in which, the hemodynamic effects were compared to metoprolol, a β1-selective adreno-receptor antagonist (6). Accordingly, loading dose of nebivolol (0.1 ml/kg) was administrated IV within the 10 min of reperfusion and continuation dose was administrated orally (2 ml/kg) by gastric gavages once daily. 

All experimental procedures were performed in accordance with the Guide for the Care and the Use of Laboratory Animals published by the US National Institutes of Health. The local animal ethic committee approval was obtained for all experimental procedure. 

***Induction of myocardial infarction***

MI was induced by the ligation of the left anterior descending coronary artery as described previously (7). The sham-control rats underwent the same procedure except ligation. All surgical procedures were performed under aseptic conditions. 

***Left ventricular function and hemodynamic parameters***

Left ventricular (LV) function was evaluated by echocardiography in lightly anesthetized animals as described previously (8). Two-dimensionally (2D)-guided M-mode echocardiography together with pulse-wave Doppler was performed using an echocardiographic system equipped with a 10 MHz sector probe (General Electric, System Five; Norway). All measurements and calculations were performed in accordance with the American Society of Echocardiography (9).

Hemodynamic measurements were performed according to our previous study (6). LV systolic (LVSP), diastolic (LVDP) and end-diastolic (LVEDP) pressures together with the maximum rise and fall rates (Δ±dp/dt) of LV pressure were recorded on a physiological recorder (10T Hardware System, PowerLab, ADI Instruments, UK) connected to the pressure transducer (MLT 0699, PowerLab, ADI Instruments, UK)

***Biochemical assessments***

Animals were anesthetized, abdomen was opened and blood samples were collected from the inferior vena cava, and the kidneys were dissected out, cleaned off the extraneous tissue and weighed. Blood samples were centrifuged and kept at -20 ^°^C until analysis. Kidneys were snap-frozen in liquid nitrogen and stored at -70 ^°^C for subsequent biochemical assays. The biochemical parameters were assayed in freshly prepared homogenates.

***Kidney functions***

As a representation of renal function, serum creatinine (Cr) and blood urea nitrogen (BUN) levels were measured by a standard technique using an Olympus AU 2700 Analyzer (Olympus Optical Co Ltd, Tokyo, Japan). 

Plasma was analyzed for C-reactive protein (CRP) in order to assess the systemic inflammation using an automated analyzer (AU 2700 Analyzer, Olympus Optical Co Ltd, Tokyo, Japan).

***Tissue total antioxidant capacity, total oxidant status and oxidative stress index***

Tissue total antioxidant capacity (TAC) and total oxidant capacity (TOC) were measured spectrophotometrically using commercially available kits according to manufacturer’s instruction (Abcam UK, Rel Assay Diagnostics Turkey). The results were expressed as nanomole Trolox equivalent/mg protein for tissue for TAC and nanomole H_2_O_2_ equivalent/milligram protein for TOC.

The percent ratio of TOC level to TAC level was accepted as the oxidative stress index (OSI) and calculated according to the following formula: (nanomole H_2_O_2_ equivalent/milligram protein)/(nanomole Trolox equivalent/mg protein) (10).

Malondialdehyde (MDA), and glutathione (GSH) levels as oxidative damage biomarker and superoxide dismutase (SOD) level as antioxidant defense system biomarker were measured using commercially available kits according to manufacturer’s instruction. 

***Tissue NO, ONO***_2_ ***- and cyclic guanylate cyclase levels***

NO was measured in the tissue supernatants as nitrite/nitrate (NOx) concentration by spectrophotometry (Roche, USA).

To assess the activation of either NO/ONO_2_- or NO/ cyclic guanylate cyclase (cGMP) pathway, ONO_2_- and cGMP levels were measured by using enzyme-linked immunoassay (ELISA) (HBT, HyCuit biotechnology, USA, Zymed Laboratories Inc., USA). Tissue protein levels were determined using the method proposed by Folin-Lowry (11). 

***Histologic assessments***

*Histochemistry*

Following the measurements of ventricular pressures, the heart and kidneys were perfused-fixed with 10% phosphate-buffered formalin at a pressure of 7.5 centimeter H_2_O for 1 hour according to our previous study (6). After fixation, tissues were excised quickly and weighed, dehydrated and embedded in paraffin.

For light microscopic evaluation, paraffin-embedded specimens were cut into 5- millimeter thick sections and stained with hematoxylin-eosin (HE) and masson-trichrome (MS). The tissues were investigated under a light microscope (Olympus BH-2). 

Severity of heart damage was examined as the early coagulative necrosis parameters (edema and heavily neutrophil infiltration and inflammation, loss of contraction bands and wavy fibers) and late signs of MI (macrophage and collagen accumulation, loss of nuclei). 

The kidneys were examined for the presence of tubular (epithelial cell detachment, atrophy, dilation, and intratubular cast formation) and glomerular (widening of the Bowman space) alterations. Severity of renal damage was scored with grading system of 0 to 3, developed by Chatterjee *et al.* (12) on a blinded basis as follows: 0=normal histology; 1=tubular cell swelling, brush border loss, nuclear condensation, with up to tubular profile showing nuclear loss; 2=as with score 1, but greater than one third and less than two thirds of tubular profile shows nuclear loss; and 3=greater than 2/3 of tubular profile shows nuclear loss.

*Immunohistochemistry*

The immunohistochemical studies were performed according to the developed technique as described previously (6). Samples were incubated using endothelial nitric oxide synthase (eNOS) Ab-1 (dilution 1:200) and inducible NOS (iNOS) Ab-1 (dilution 1:200) antibodies (Abcam UK) for 2 hr at room temperature and neuronal NOS (nNOS) (dilution1: 100) (Abcam UK) for overnight at 4 ^°^C according to the manufacturer instructions. Sections were examined under light microscope and labeling intensity was graded using a semi-quantitative scale of 1+ (weak), 2+ (moderate) or 3+ (strong) labeling on a blinded basis.

***Statistical analysis***

Statistical analysis was performed with SPSS 24.0 software program (Chicago, IL, USA). All variables were expressed as mean±standard deviation. Echocardiographic, hemodynamic and biochemical measurements were analyzed by ANOVA and *post-hoc* Bonferroni test and *P*<0.05 was considered statistically significant.

## Results

A total of 54 animals were included in the study. MI was confirmed by the increase in troponin T levels. However, troponin T levels were insignificant between groups (2.13, 2.26 and 2.35 nanogram/milliliter for MI-control, MI-neb1 and MI-neb2 groups, respectively; *P*>0.05). 

There was no death in the sham-control group. Mortality rates were 11 %, 5% and 5% for MI-control, MI-neb1 and MI-neb2 groups, respectively. Death was observed within the first 24 hr of infarction in infarct groups. 

***LV structure and functions***

**Figure 1:**
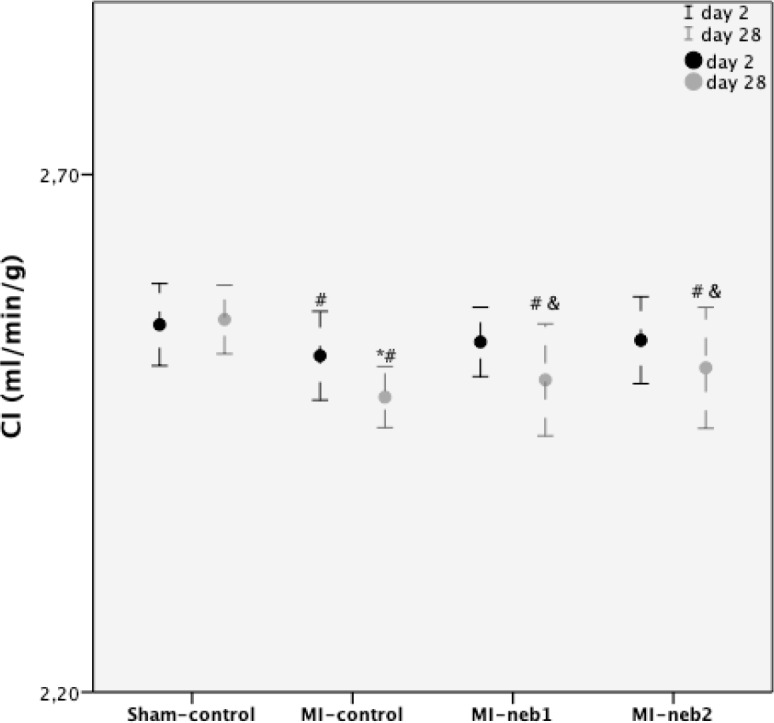
Left ventricular functions characterized by cardiac index (CI)

Compared to sham-control, LV structural changes (characterized by increased LVEDd, and LVEDv) and functional abnormalities (characterized by decreased EF and CO) were significant in MI-control group as early as 2 day of MI (*P*<0.05 for all comparisons). This trend continued for 28 days (Table 1). Compared to MI-control, LV function characterized by cardiac index (CI) was maintained in nebivolol-treated groups (Figure 1). 

***Hemodynamic parameters***

Hemodynamic parameters are shown in Table 2. MI-control group was characterized by increased mean blood pressure (MBP) and LVEDP together with decreased Δ± dp/dt. Nebivolol treatment slightly but not significantly lowered the MBP. The increase in LVEDP and the decrease in Δ± dp/dt were prevented by nebivolol. The differences in MBP, LVEDP and Δ± dp/dt were not statistically significant between MI-neb1 and MI-neb2 groups (*P*>0.05) (Table 2). 

***Biochemical assessments***

In the early period of MI compared to the sham-control, the increase in BUN and plasma Cr was not statistically significant in MI-control group. However, this increase was significant in the late period of MI (28th day). In this group, plasma CRP levels were also significantly higher than sham-control animals (*P*=0.001, for all comparisons) (Table 3). 

**Figure 2:**
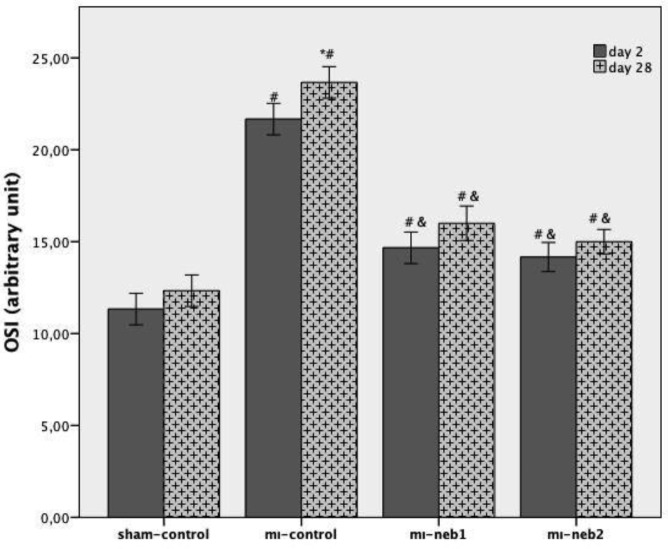
Oxidative stress status of the groups (OSI)

Compared to sham-control, MI-control group was characterized by increased TOC, decreased TAC levels and subsequent increased OSI. Nebivolol treatment prevented the increase in OSI (*P*<0.003). Although the OSI of MI-neb2 was lower than of MI-neb1, this decrease was not reached the statistically significant level (*P*>0.05) (Figure 2).

Induction of MI caused decrease in SOD, and GSH and increase in MDA levels of MI-control animals (*P*<0.05 for all comparisons). This trend continued throughout the study period. Nebivolol treatment prevented the decrease in SOD and GSH and increase in MDA levels (*P*<0.05 for all comparisons). There was no statistically significant difference between MI-neb groups (*P*>0.05) (Table 4)

Although tissue NOx/ONOO- levels were increased early after MI, cGMP levels were not changed. Nebivolol treatment prevented the increase in NOx and ONOO- levels. The differences in the tissue NOx and ONOO- levels between the MI-neb1 and MI-neb2 groups were not statistically significant in the early period of MI (2nd day of MI). However, this difference reached the statistically significant level at the late period of MI (28 ^th^ day of MI) (Table 4)

***Histologic assessments***

**Figure 3:**
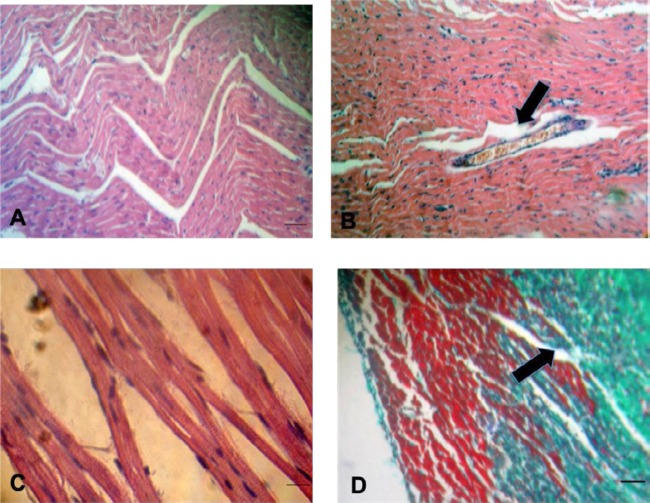
Light micrographic features of myocardial infarction (x400)A) coagulative necrosis characterised by wavy myofibrils together with neutrophil infiltration B) polymorphous nuclear leukocyte infiltration into the infarct area and significant leukocyte infiltration into the vasculature (black arrow) C) loss of condensation bands D) collagen accumulation (black arrow)

The light microscopic images of sham-control rats showed normal myocardium, which demonstrated the normal orderly arrangement of myocytes and scant interstitial fibrosis. MI-control group was characterized by coagulative necrosis followed by typical inflammatory response and repair. The changes of coagulative necrosis become evident early after MI (2 day of MI). Wavy cardiac muscle cells represented the coagulative necrotic cells and the presence of neutrophils represented that the inflammation could be recognized on HE-stained slices of MI-control rats. Collagen accumulation was also observed in MS-stained sections too (Figure 3) 

**Figure 4:**
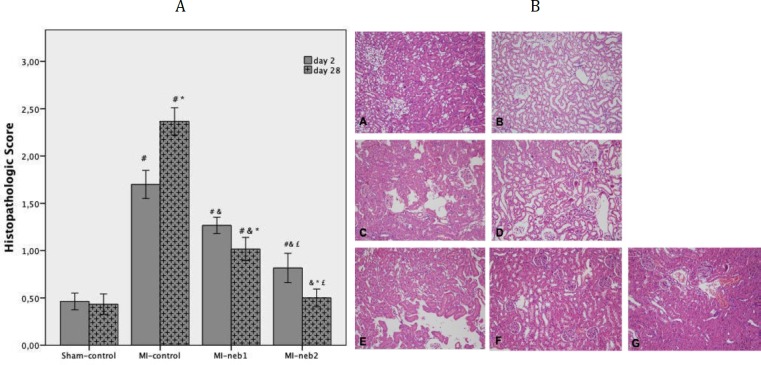
(A) Histopathological scores of renal tissue samples. Results are presented, as mean±SD. In myocardial infarction (MI)-control group, renal damage was significant in both periods of MI. Compared to the MI-control group; histopathological scores were significantly lower in nebivolol-treated groups. (*P<*0.05 ^*^ compared to day 2, ^#^ compared to sham-control group at same point in time and compared to MI-control group at same point in time, ^£ ^compared to MI-neb1 group at same point in time)

Histopathological evaluation revealed that the renal tissues of the sham-control group had normal structure with no pathological changes. In the MI-control group, focal tubular damage characterized by focal brush border loss, tubular cell swelling, nuclear condensation and loss was prominent in the early period of MI (2 day of MI). Extensive tubular damage characterized by flattening in epithelial cells, and total brush border loss in proximal tubules was recognized in the late period of MI (28 day of MI) in this group. Compared to MI-control, regeneration signs (especially reappearance of brush borders) were observed in nebivolol-treated groups. These changes were more prominent in MI-neb2 group. Especially at the late period of MI (28 day of MI), renal tissues of MI-neb2 animals were almost same as sham-control rats (Figure 4).

**Figure 5:**
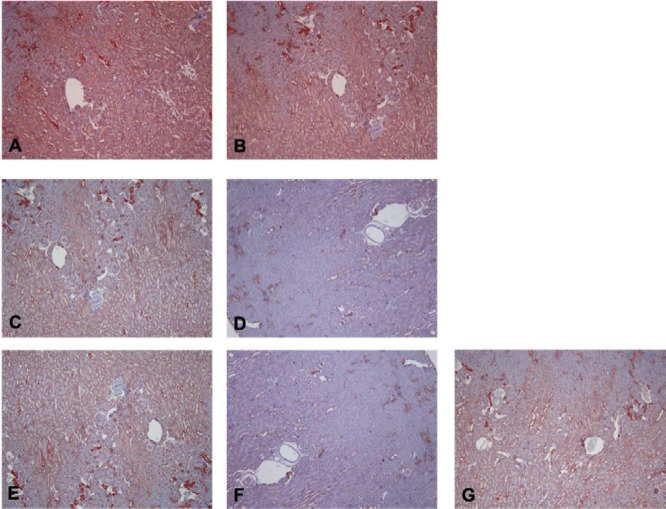
Examples of histological sections of kidney reacted to inducible nitric oxide synthase (iNOS) antibody (x100)

Immunolabeling intensities of all groups are given in Table 5. In sham-control group, strong nNOS together with moderate eNOS and iNOS immunoreactivity were observed throughout the study. Cytoplasmic iNOS immunoreactivity was observed both in the cortex and proximal tubules. Moderate iNOS staining was observed in sham-control group throughout the study (labeling intensity 2+ for both periods of MI) (Figure 5). In MI-control group, iNOS immunolabeling intensity was increased early after MI. This trend was continued throughout the study (labeling intensity 3+ for both periods of MI) (Figure 5). In the nebivolol-treated groups, iNOS immunolabeling intensities were similar to sham-control at the early period of MI (2^nd^ day of MI). However, at the late period of MI (28th day of MI), iNOS-labeling intensities were weaker than sham-control (labeling intensity 1+ for both MI-neb groups) in these groups. 

**Figure 6:**
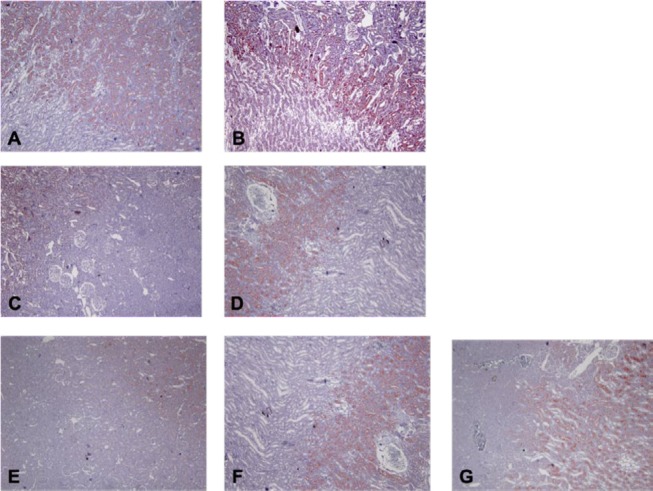
Examples of histological sections of kidney reacted to endothelial nitric oxide synthase (eNOS) antibody (x100)

Cytoplasmic-membranous eNOS immunoreactivity both in juxtamedullary region and brush borders of proximal tubules was observed. Moderate eNOS staining was observed in sham-control group throughout the study (labeling intensity 2+ for both periods of MI) (Figure 6). Although eNOS staining intensity was same as sham-control at the early period of MI (labeling intensity +2) in MI-control, this was stronger than sham-control at the late period of MI (28th day of MI). Weak labeling intensity was observed at the early period of MI in the nebivolol-treated groups (0-1+). eNOS immunolabeling intensities were same as sham-control at the late period of MI for these groups too (Figure 6).

**Figure 7:**
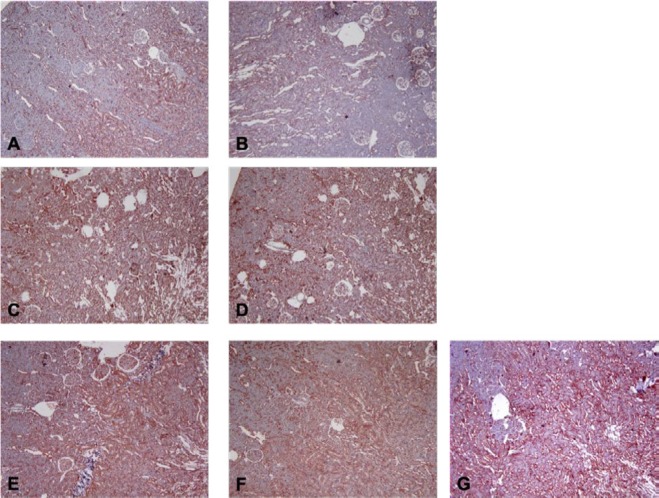
Examples of histological sections of kidney reacted to neuronal nitric oxide synthase (nNOS) antibody (x100). Red colouring indicates positive immunostaining. The individual panels represent kidney sections from myocardial infarction (MI)-control animals at the A) 2^nd^ and B) 28^th^ days; from MI-neb1 at the D) 2^nd^ and E) 28^th^ days; from MI-neb2 at the E) 2^nd^ and F) 28^th^ days and G) sham-control animals

Cytoplasmic and partly nuclear nNOS immunolabeling in juxtamedullary region was observed. Strong nNOS immunoreactivity was observed in sham-control through the study (labeling intensity 3+ for both periods of MI). nNOS immunoreactivity was decreased early after MI in MI-control group. This decrease was continued throughout the study (labeling intensities were +2 and 1+ for the 2^nd^ and 28^th^ day of MI respectively). In nebivolol-treated groups, nNOS immunolabeling intensities were same as sham-control for both periods of MI (Figure 7).

## Discussion

***MI-caused heart failure with preserved ejection fraction (HFpEF)***

Bidirectional communication between the heart and kidney is well known. Under normal circumstances, this communication coordinates the modulation of the cardiac output, vascular tone and volume status and excretion of metabolic waste products (13). Disruption of one of these pathways causes cardiac and/or renal dysfunction and failure of one organ accelerating the damage and failure of the other, which is defined as CRS (14).

Preliminary studies on the pathophysiology of CRS have shown that heart disease leads to nephropathy, and relevant studies have supported the renal venous hypertension as the main mechanism. Although the decrease in cardiac stroke volume is another important factor, in patients with heart failure with or without systolic dysfunction the disruption of renal function in similar proportions and the development of CRS in acute heart failure with hypertension suggest that renal venous congestion is more important than renal hypoperfusion in the development of CRS and constitutes the main pathophysiological mechanism (15). In our study, the reduction in EF after MI was mild. The significant increase in LVEDP, despite the protection of LV systolic function, is in line with the HFpEF model (16). Although we observed a significant decrease in MBP after MI, this decrease is within physiological limits and does not reflect a significant hypotension (17). Our results are in line with Cho *et al.* (18) and suggest that renal hypoperfusion is not a primary factor in the development of CRS. 

***Inflammation could be a connector between the heart and kidney***

Although mechanisms of renal venous congestion leading to renal dysfunction is still unclear, mechanical pressure effect of renal venous hypertension on distal tubules, increased renal interstitial pressure and the activation of the sympathetic and/or RAAS are considered as possible mechanism (19, 20). Among these mechanisms, activated RAAS and oxidative stress as a response to up-regulated inflammatory status are found to be the key mechanisms in recent preclinical studies (21). Parallel with these preclinical studies, release of inflammatory mediators in patients with peripheral congestion was also shown (22). Theoretically, renal congestion increases luminal pressure via causing tubular compression and by this way decreases transglomerular pressure and glomerular filtration rate (GFR) (23, 24). In contrast to theoretical knowledge, retrogradely transduced glomerular hypertension without an impact on GFR was shown on abdominal venous congestion model in recent studies (25). In related studies, mechanism is explained as compensatory increase in intraglomerulary tension via afferent vasodilation and efferent vasoconstriction at a single nephron in order to increase the GFR, thereby developing hyperfiltration as a response to intraglomerular hypertension. Researchers also showed the inflammation as a key contributor for the worsening of kidney function. In our study, subclinical AKI findings together with glomerular enlargement (an indicator of intraglomerular hypertension) in histological slices in the early period of MI support this hypothesis (25). Moreover, systemic inflammation as indicated by increased plasma CRP levels suggests that inflammation could be a key connector between the heart and kidney crosstalk. 

The tubular system, especially proximal tubules are known to play an important role both in AKI and long-term regulation of renal function, especially GRF (26). In fact, tubular damage has been shown as an early and decisive step in many AKIs (27, 28). Accordingly during the tubular injury, proximal tubular cells redifferentiate in order to replacement of lost epithelial cells, but according to the Venkatachalam *et al.*, during this process some cells fail to redifferentiate and continue to produce factors that stimulate proliferation, which may lead to fibrosis (29). In addition, Choe *et al.* showed that inflammation developed after MI caused long-term chronic kidney damage by causing interstitial fibrosis (18). In a recent study, by analyzing the kidney biopsies of renal transplant recipient patients to investigate the correlation between the morphological features of the injury and the functional outcomes (30), a correlation was found between the degree of epithelial cell pkynosis, flattening and brush border loss and the severity of the renal dysfunction. In our study, focal tubular damage characterized by the loss of brush border in histological sections in the early period after MI was replaced by total tubular damage characterized by loss of total brush border and interstitial fibrosis. In line with these morphological features, subclinical functional damage in the early phase was transformed into chronic renal dysfunction characterized by increased BUN and Cr levels in the late period. 

***Inflammation-induced nitrosative stress was the responsible mechanisms for renal damage***

Physiological levels of ROS and RNS are required to perform a normal cellular function (31). Thus in tubules, NO, O_2_−, and ONO_2_− regulate water reabsorption to preserve electrolyte homeostasis and extracellular fluid volume (32). According to the body of evidence, NO has biphasic action on sodium (Na) transport in proximal tubules depending on its concentration. While the low concentration of NO inhibits the transport via activation of Na/K-ATPase activity in cGMP dependent pathway (32), the high concentration of NO stimulates transport by inhibition of Na/K-ATPase (34). However, according to Guzman *et al.*, this action is due to ONO_2_− instead of NO itself (35). Furthermore, recent studies have shown that in this biphasic effect, the source of NO was different (32). While in the physiological conditions, low level NO produced by nNOS acts as an autacoid and reduces fluid and Na reabsorption (36), high level of NO produced by iNOS (especially with cytokine induction) in tubules increases fluid and Na reabsorption (35). In the light of this knowledge, the increased iNOS immunoreactivity in proximal tubules (3+ for MI-control group, for both periods of MI) together with increased OSI and high renal ONO_2_− levels suggest that oxidative stress induced-iNOS is the source of high NO in both periods of MI. Parallel with these results, we observed decrease in nNOS immunoreactivity in MI-control group with time dependent manner (2+ and 1+ for 2nd and 28th day of MI, respectively). As discussed earlier, in order to increase the GFR, afferent vasodilation together with efferent vasoconstriction occurs as a compensatory mechanism in response to venous congestion. According to recent study on a rat spontaneous hypertension model, this compensatory mechanism is regulated by nNOS, and while SOD activates the effect of nNOS on afferent arterioles, O_2_− inhibits the regulatory effect of nNOS on the afferent arterioles (37). In our study, despite the tubular damage findings, the maintenance of functional capacity in the early period of MI supports these results. 

It was known that ROS and RNS redox disequilibrium developed through different mechanisms that stimulate structural and functional abnormalities leading to cell injury (38-40). In addition to cell injury, ROS production can lead to a vicious circle of ROS-induced ROS release, which can be explained by further releasing of ROS from the mitochondria as a result of ROS-related dysfunction (41). Indeed, Quoilin *et al*. demonstrated that cytokine-induced iNOS overexpression induced the formation of intracytosolic O_2_- and NO radicals (ŸNO) and that these simultaneously produced radicals caused the formation of ONO_2_− by competing with SOD in the cytosol. In the same study, researchers also showed that ROS and RNS overproduction caused further mitochondrial damage by further disrupting cellular ATP production and emphasized the basic mechanism of tubular damage as a mitochondrial-induced cytopathic hypoxia (42). Moreover, Branders and Halliwell showed the mitochondrial damage mechanism caused by O_2_- as an opening of ATP-sensitive K+ channels (43) and mitochondrial membrane lipid peroxidation (44). In our study, high renal MDA levels with increased OSI supports the mechanism of mitochondrial membrane lipid peroxidation. 

Is it well recognized that the most important regulatory mechanism in kidney is the glomerulo-tubular balance (GTB), which adjusts absorption of salt and water in proximal tubules in proportion to changes in GFR (45). Unlike previous studies, in recent studies, it was shown that flow-dependent Na+ and HCO_3_- transport in proximal tubules is regulated by Na+/H+ exchanger isoform-3, and independent from the neuronal and systemic hormonal regulation (46). Brush border microvilli function as flow sensor in the proximal tubules (47). In the tubular sections, eNOS activity was shown in the proximal tubule, medullary thick ascending limb of the loop of Henle (mTAL) and collecting duct (48, 49). Moreover flow-induced eNOS translocation in TAL was also reported. In our study, observation of increase in eNOS immunoreactivity both in the brush border of proximal tubules and juxtamedullary region (these nephrons are characterized by long loop of Henle) in the late period of MI supports these results and emphasizes the role of eNOS in tubular damage in the late period of MI.

***The*** ***beneficial effects of nebivolol were closely related to the reduction of nitrosative damages as well as hemodynamic alterations***

A number of drugs, such as RAAS antagonists, vasodilators, vasopressin, adenosine antagonists, inotropes, nesiritide (recombinant form of B-type natriuretic) and diuretics have been studied in the treatment of CRS. Some of the studies in which heterogeneous results were obtained (RAAS antagonists, diuretics, and beta-blockers) reported positive results on heart failure; however, a stable improvement in the prognosis of nephropathy could not be provided. The main target in these studies was usually hemodynamic parameters and neurohumoral activation. In addition to preclinical observations, clinical trials with diuretics and vasodilators have failed to record improvements in the nephropathy parallel to the improvement of hemodynamic parameters. Furthermore, the tendency of nephropathy to worsen with intensive diuretics and RAAS antagonist treatments indicates the necessity of different mechanisms in treatment. Strategies to reduce inflammation and oxidative stress are limited. To the best of our knowledge, this is the first study investigating the NO-mediated effects of nebivolol on both Type I and Type II CRS in a rat model of MI. 

According to our results, significant improvements were achieved in hemodynamic parameters with nebivolol. In the nebivolol groups, EF significantly improved compared to the MI-control. However, the improvement in LVEDP provided by nebivolol was much more pronounced. Despite the effective reduction in LVEDP with nebivolol in both periods of MI, the mean values ​​were still significantly higher compared to the sham-control group. Thus, a partial improvement with nebivolol may be mentioned, especially in diastolic functions. On the other hand, the effect of nebivolol on oxidative stress parameters was stable and evident. Nebivolol effectively prevented both subclinical and clinical nephropathy developed after MI. Contrary to significantly elevated Cr levels in MI-control group in the late period of MI (1.4 milligram/deciliter), unchanged Cr levels in nebivolol-treated groups in both periods of MI (0.9 milligram/deciliter) support this result. It should also be emphasized that the nebivolol dose used in this study was the minimum beta-blocker dose (6). 

***The NO-mediated effects of nebivolol based on its source can be summarized as***

*1. Prevention of oxidative/nitrosative damage by decreasing iNOS activity*

As discussed earlier, NO, O_2_−, and ONO_2_− regulate water reabsorption in tubules (32). While low level of NO produced by nNOS reduces fluid and Na reabsorption (36), high level of NO produced by iNOS increases fluid and Na reabsorption. Moreover, it is known that mitochondria is the main source of ROS and increased ROS production leads to production of more ROS resulting from the mitochondrial dysfunction, which results in a vicious circle of -ROS induced ROS release- (50). In the case of oxidative stress, the reaction of NO with ROS can produce the ONO_2_-. In our study, compared to the MI-control animals, decreased OSI, NOx and ONO_2_- levels together with the increased SOD levels in nebivolol-treated animals shows that nebivolol decreases the oxidative/nitrosative damage in kidney after MI. Parallel with this findings, decreased iNOS immunoreactivity in these groups in both periods of MI supports the hypothesis that the source of high level of NO in the kidney is the iNOS activation, and nebivolol decreases this high level of NO through the iNOS inhibition. 

*2. Preservation of nNOS activity in order to maintain GFR*

It was shown that NO produced by nNOS acts as a compensatory mechanism and causes afferent vasodilation together with efferent vasoconstriction in order to increase GFR, and this is blocked by superoxide anions (37). In our study, prevention of high levels of NO production via inhibition of iNOS protects the physiological level of NO and maintains GFR. Similar nNOS immunoreactivity levels with sham-control animals in nebivolol-treated groups and maintenance of renal functional capacity in both periods of MI supports this hypothesis. 

*3. Restoration of eNOS activity in the late period of MI:*

The role of eNOS in the Na+ and HCO3- transport in proximal tubules is known (46-49). It is also known that production of high level of NO by up-regulated iNOS neutralizes the cysteine residues of arginase. This increases the arginase activity, which results in L-arginine consumption. Reduced availability of L-arginine induces eNOS uncoupling, and eNOS becomes a radical generating enzyme. As a result, NO directly reacts with superoxide anions to form ONO_2_-, which is responsible for nitrosative damage (51). In our study, decreased eNOS immunoreactivity in the early period of MI together with restored eNOS activity in the late period in nebivolol-treated groups supports the hypothesis that decreasing high level of NO by nebivolol via inhibition of iNOS restores the eNOS activity in the late period of MI.

**Table 1:** . Left ventricular structure and functions of the groups

**Parameter/Time**	**Sham-control** **(n=18)**		**MI-control** **(n=18)**		**MI-neb 1** **(n=18)**		**MI-neb 2** **(n=18)**	
	**day 2**	**day 28**	**day 2**	**day 28**	**day 2**	**day 28**	**day 2**	**day 28**
**LVEDd (cm)**	0.67±0.09	0.68±0.04	0.67±0.07	0.74±0.10^#^	0.65±0.06	0.69±0.02^&^	0.62±0.04	0.60±0.02^&^
**LVEDv (ml)**	0.70±0.04	0.66±0.05	0.78±0.06^#^	0.81±0.04^#^	0.74±0.12	0.77±0.06^#^	0.71±0.09	0.72±0.04^#^
**EF (%)**	70.17±6.20	69.50±6.20	58.17±7.90^#^	56.00±6.79^#^	66.17±3.98^#&^	65.60±2.79^#&^	64.28±2.18^#&^	63.60±2.39^#&^
**CO (ml/min)**	752±269	742±149	726±210	797±197	765±214	752±220	759±236	768±213

*P<*0.05 #compare to sham-control group at same point in time and compare to MI-control group at same point in time

LVEDd: Left ventricular end-diastolic dimension; LVEDv: Left ventricular end-diastolic volume; EF: Left ventricular ejection fraction; CO: Cardiac output; MI: Myocardial infarction

CI of myocardial infarction (MI)-control group was significantly higher than the sham-control group for both periods MI; however, CI was maintained in nebivolol-treated groups (*P<*0.05 ^* ^compared to day 2, ^# ^compared to sham-control group at same point in time and compared to MI-control group at same point in time, ^£ ^compared to MI-neb1 group at same point in time)

**Table 2:** Hemodynamic parameters measured in the groups

**Parameter/Time**	**Sham-control** **(n=6)**		**MI-control** **(n=6)**		**MI-neb 1** **(n=6)**		**MI-neb 2** **(n=6)**	
	**day 2**	**day 28**	**day 2**	**day 28**	**day 2**	**day 28**	**day 2**	**day 28**
**MBP (mmHg)**	109.1±7.4	110.1±6.7	126.58.8^#^	132.8±8.3^#^	90.310.0^#^	95.0±8.1^#^	93.27.8^#^	96.2±8.2^#^
**LVEDP (mmHg)**	2.0±0.23	2.3±0.3	28.3±4.1^#^	33.7±1.7^#^	12.5±4.0^#^^&^	15.2±3.6^#^^&^	14.3±2.8^#^^&^	17.1±1.5^#^^&^
**Δ+dp/dt (mmHg/min)**	6716±574	6600±352	4078±411^#^	3831±398*^#^	4613±291^#,&^	4765±278^#& ^	4523±186^#,&^	4621±236^#& ^
**Δ+dp/dt** **(mmHg/min)**	5308±595	5058±618	2691±346^#^	2861±268*^#^	3354±339^#,&^	3410±258^#^^&^	3028±126^#,&^	3215±224^#,&^

*P<*0.05 ^*^ compare to day 2, ^# ^compare to sham-control group at same point in time and compare to MI-control group at same point in time

MBP: Mean blood pressure; LVEDP: Left ventricle end-diastolic pressure; D+dp/dt: Maximum rise of left ventricle pressure

D–dp/dt: Maximum fall of left ventricle pressure; MI: Myocardial infarction

**Table 3:** Kidney functions measured in the groups

**Parameter/Time**	**Sham-control** **(n=6)**		**MI-control** **(n=6)**		**MI-neb 1** **(n=6)**		**MI-neb 2** **(n=6)**	
	**day 2**	**day 28**	**day 2**	**day 28**	**day 2**	**day 28**	**day 2**	**day 28**
**BUN ** **(mg/dl)**	16.28±0.98	15.57±0.67	24.67±1.18 ^#^	48.20±6.67* ^#^	21.34±2.10 ^#&^	25.37±3.67^#&^	19.42±4.63 ^#&^	22.67±4.73^#&^
**Cr (mg/dl)**	0.84±0.08	0.77±0.03	0.73±0.04 ^#^	1.40±0.09* ^#^	0.70±0.02^#&^	0.94±0.03 ^#&^	0.73±0.02 ^#&^	0.87±0.04^#&^

*P<*0.05 * compare to day 2, ^#^ compare to sham-control group at same point in time and compare to MI-control group at same point in time

BUN: Blood urea nitrogen; Cr: Creatinine; CRP: C-reactive protein; MI: Myocardial infarction

Myocardial infarction (MI)-control group was characterized by increased OSI. Nebivolol treatment prevented the increase in OSI. Although the OSI of MI-neb2 was lower than of MI-neb1, this decrease was not reached the statistically significant level (*P<*0.05 ^*^ compared to day 2, ^# ^compared to sham-control group at same point in time and compared to MI-control group at same point in time)

**Table 4:** Tissue oxidative/nitrosative damage and antioxidant capacity of the groups

**Parameter/Time**	**Sham-control** **(n=6)**		**MI-control** **(n=6)**		**MI-neb 1** **(n=6)**		**MI-neb 2** **(n=6)**	
	**day 2**	**day 28**	**day 2**	**day 28**	**day 2**	**day 28**	**day 2**	**day 28**
**SOD (U/mg protein)**	5.29± 0.4	5.08± 0.2	3.11±0.9^#^	1.86±0.5^#,*^	4.83±0.3^&^	4.75± 0.4^#,&^	4.37± 0.7^&,£^	4.46± 0.6^¥,&,£^
**MDA** **(pmol/g tissue)**	0.42±0.26	0.39±0.15	0.78±0.16 ^#^	0.89±0.20 ^#^	0.63±0.18 ^#&^	0.58±0.22 ^#&^	0.55±0.17 ^#&^^£^	0.48±0.12 ^#&^^£^
**NOx** **(nmol(mg protein)**	145±7.2	150±10.5	348±22.3^#^	373±18.3^#^	251±13.1^#,&^	188±11.6^#,&,*^	271±16.9^#,&^	232±14.1^#,&,£,*^
**ONO**_2_^-^ **(nmol/g tissue)**	114±3.1	125±6.2	308±18.2^#^	366±12.6^#,*^	286±22.4^#&^	250±32.3^#,&,*^	234±24.9^#,&^	264±29.8^#,&,*^

*P<*0.05 ^*^ compared to day 2, ^# ^compared to sham-control group at same point in time and compared to MI-control group at same point in time, ^£ ^compared to MI-neb1 group at same point in time

SOD: Superoxide dismutase; MDA: Malondialdehyde; NOx: Nitrite/nitrate; ONO2-: Peroxynitrite; MI: Myocardial infarction

**Table 5:** Immunolabeling intensities of the groups in the histopathological assessmnet

**Parameter/Time**	**Staining and localization**	**Labeling Intensity **							
		**Sham-control**		**MI-control**		**MI-neb 1**		**MI-neb 2**	
		**day 2**	**day 28**	**day 2**	**day 28**	**day 2**	**day 28**	**day 2**	**day 28**
**eNOS**	Cytoplasmic staining in brush borders and juxtamedullary region	2+	2+	2+	3+	0-1+	2+	0-1+	2+
**nNOS**	Cytoplasmic and nuclear staining in juxtamedullary region	3+	3+	2+	1+	3+	3+	3+	3+
**iNOS**	Cytoplasmic staining in cortex and proximal tubules	2+	2+	3+	3+	2+	1+	2+	1+

Labeling intensities: 1+: weak 2+: moderate 3+: strong

eNOS: Endothelial nitric oxide synthase; iNOS: Inducible nitric oxide synthase; nNOS: Neuronal nitric oxide synthase; MI: Myocardial infarction

(4B) Light micrographic features of kidneys (x200). A) focal tubular damage characterized by focal brush border loss, tubular cell swelling, nuclear condensation and loss B) extensive tubular damage characterized by flattening in epithelial cells, total brush border loss in proximal tubules C-E) focal regeneration findings with reappearance of brush borders D-F) extensive regeneration findings with almost normal tubular structure G) normal tubular structure. The individual panels represent kidney sections from myocardial infarction (MI)-control animals at the A) 2^nd^ and B) 28^th^ days; from MI-neb1 at the C) 2^nd^ and D) 28^th^ days; from MI-neb2 at the E) 2^nd^ and F) 28^th^ days and G) sham-control animals

Red colouring indicates positive immunostaining. The individual panels represent kidney sections from myocardial infarction (MI)-control animals at the A) 2^nd^ and B) 28^th^ days; from MI-neb1 at the D) 2^nd^ and E) 28^th^ days; from MI-neb2 at the E) 2^nd^ and F) 28^th^ days and G) sham-control animals

Red colouring indicates positive immunostaining. The individual panels represent kidney sections from myocardial infarction (MI)-control animals at the A) 2^nd^ and B) 28^th^ days; from MI-neb1 at the D) 2^nd^ and E) 28^th^ days; from MI-neb2 at the E) 2^nd^ and F) 28^th^ days and G) sham-control animals

***There was no difference between two nebivolol treatment regimes***

NO is a weak RNS and induces the production of ONO_2_- and other highly oxidizing ROS (5). Therefore, in addition to the beta-blocker activity of nebivolol, NO-mediated effects are quite valuable in both types of CRS (Type I and II), in which early and late oxidative damage is prominent***. ***When the effects of different nebivolol treatment regimes on renal injury was assessed, compared to MI-neb1 group renal ONO_2_- and OSI levels were lower in MI-neb2 group. However, this did not reach the statistically significant level. Parallel to the biochemical results, regeneration findings were more prominent in the HE-stained sections of this group, but none of the three isoforms of NOS immunoreactivity levels differed between the groups. The possible reason for this is the semi-quantitative measurement of NOS levels in this study. Although we showed positive effects of early nebivolol administration (within 10 minute of MI by IV) on LV dysfunction in a rat MI model in our previous studies (5, 52), in this study early nebivolol administration had a similar effect with delayed administration of nebivolol (daily oral administration) on both types of CRS. The possible reason is the induction of compensatory mechanisms in order to preserve renal function in the early period after MI. Therefore, there is a need for studies with a longer study period.

## Conclusion

In this study, heart failure developed by MI is in line with the HFpEF model. Subclinical acute renal damage developed in the early period of MI transformed into renal failure in the late period, which was consistent with Type I and Type II CRS, respectively. Nebivolol treatment prevented the both types of CRS developed after MI. Inflammation-induced nitrosative stress is the major mechanism of renal damage, and beneficial effects of nebivolol were closely related to the reduction of nitrosative damage as well as hemodynamic alterations. Moreover, NO-mediated mechanisms that contribute these effects can summarize as: 1) prevention of oxidative/nitrosative damage by decreasing iNOS activity; 2) preservation of nNOS activity in order to maintain GFR; 3) restoration of eNOS activity in the late period of MI.
